# Highly Anti-Markovnikov Selective Oxidative Arene Alkenylation Using Ir(I) Catalyst Precursors and Cu(II) Carboxylates

**DOI:** 10.1021/acs.organomet.4c00030

**Published:** 2024-03-20

**Authors:** Hannah Ketcham, Weihao Zhu, T. Brent Gunnoe

**Affiliations:** Department of Chemistry, University of Virginia, Charlottesville, Virginia 22904, United States

## Abstract

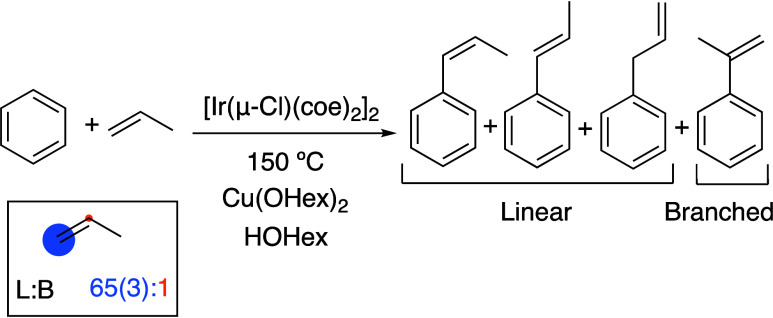

The Ir(I) complex [Ir(μ-Cl)(coe)_2_]_2_ (coe = *cis-*cyclooctene) is a catalyst precursor for benzene alkenylation using Cu(II) carboxylate salts. Using [Ir(μ-Cl)(coe)_2_]_2_, propenylbenzenes are formed from the reaction of benzene, propylene, and CuX_2_ (X = acetate, pivalate, or 2-ethylhexanoate). The Ir-catalyzed reactions selectively produce anti-Markovnikov products, *trans*-β-methylstyrene, *cis*-β-methylstyrene, and allylbenzene, along with minor amounts of the Markovnikov product, α-methylstyrene. The selectivity for the anti-Markovnikov products changed as the reaction progressed. For example, in a reaction that uses 240 equiv of Cu(OHex)_2_ (related to Ir), the selectivity for the anti-Markovnikov products increases from 18:1 at 3 h to 42:1 at 42 h with 30 psig of propylene at 150 °C. Studies of product stability have revealed that the increase in the selectivity for anti-Markovnikov products is not the result of an isomerization process or the selective decomposition of specific products. Rather, the change in selectivity correlates with the ratio of Cu(II) to Cu(I) in the solution, which decreases as the reaction progresses. We propose that the identity of the active catalyst changes as Cu(I) is accumulated, resulting in the formation of an active catalyst that is more selective for anti-Markovnikov products. Using a 4:1 Cu(I)/Cu(II) ratio at the start of the reaction, a 65(3):1 anti-Markovnikov/Markovnikov ratio is observed.

## Introduction

**Scheme 1:**
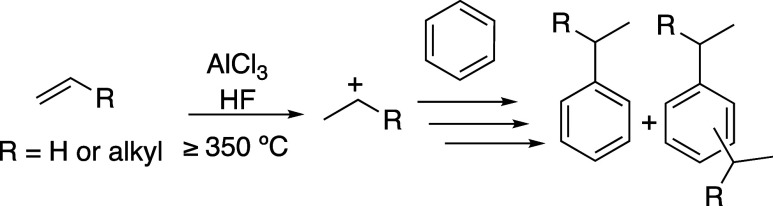
Traditional Friedel–Crafts Catalyzed Arene Alkylation When using α-olefins, acid-catalyzed arene alkylation reactions are selective for Markovnikov (branched) products.

Alkyl and alkenyl arenes are precursors to commercial products including pharmaceuticals, plastics, and detergents.^1−3^ Alkyl arenes can be produced from arenes and olefins using acid-catalyzed reactions such as Friedel–Crafts alkylation or the use of acidic zeolite catalysts.^4^ The mechanism of acid-catalyzed arene alkylation is generally proposed to operate through olefin protonation, followed by an electrophilic aromatic substitution (Scheme 1). When using α-olefins, such as propylene, the intermediate carbocation formed from olefin protonation dictates the formation of Markovnikov products.^5,6^ Herein, Markovnikov products are termed branched products, and anti-Markovnikov products are termed linear products (see below for more specific definitions for products of benzene and propylene). In addition, alkylated arenes are typically more reactive than the starting arene, which results in the formation of polyalkylated arenes, even at low arene conversion for some processes.^7^ As a result, energy-intensive distillation and transalkylation steps are often required to increase the yield of the monoalkylated arene.^8,9^

**Scheme 2:**
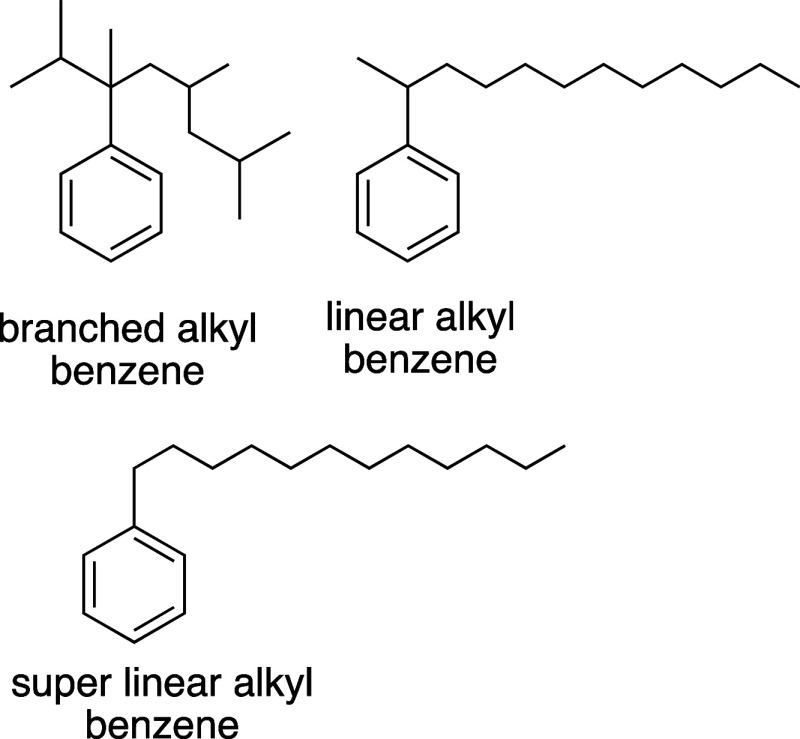
Branched Alkyl Benzene, Linear Alkyl Benzene, and Super Linear Alkyl Benzene

Highly branched alkyl benzenesulfonates (i.e., branched alkyl benzenesulfonates) were used as detersive agents until the 1960s, but they were discontinued due to biodegradability issues.^10,11^ Linear alkyl benzenes (LABs), which are straight chain alkanes with *n*-substituted (*n* > 1) phenyl groups, are used to synthesize linear alkyl benzenesulfonates, which are modern detersive agents.^12^ The use of 1-phenyl alkanes as surfactant precursors could offer useful properties as they possess the same straight chain linearity as LABs and natural soap precursors such as stearic acid and lauric acid.^13^ Previous studies have shown that surfactants made from linear 1-phenyl alkanes might offer enhanced detersive properties compared to linear 2- and 3-phenyl alkanes.^14^ To differentiate internally substituted LABs from 1-phenyl alkanes, our group has previously coined the term super linear alkyl benzenes (SLABs, Scheme 2) for 1-phenyl alkanes.^15,16^ SLABs can be synthesized through a Friedel–Crafts acylation and Clemmensen reduction, but this strategy is not viable for scaled production.^1^

**Scheme 3:**
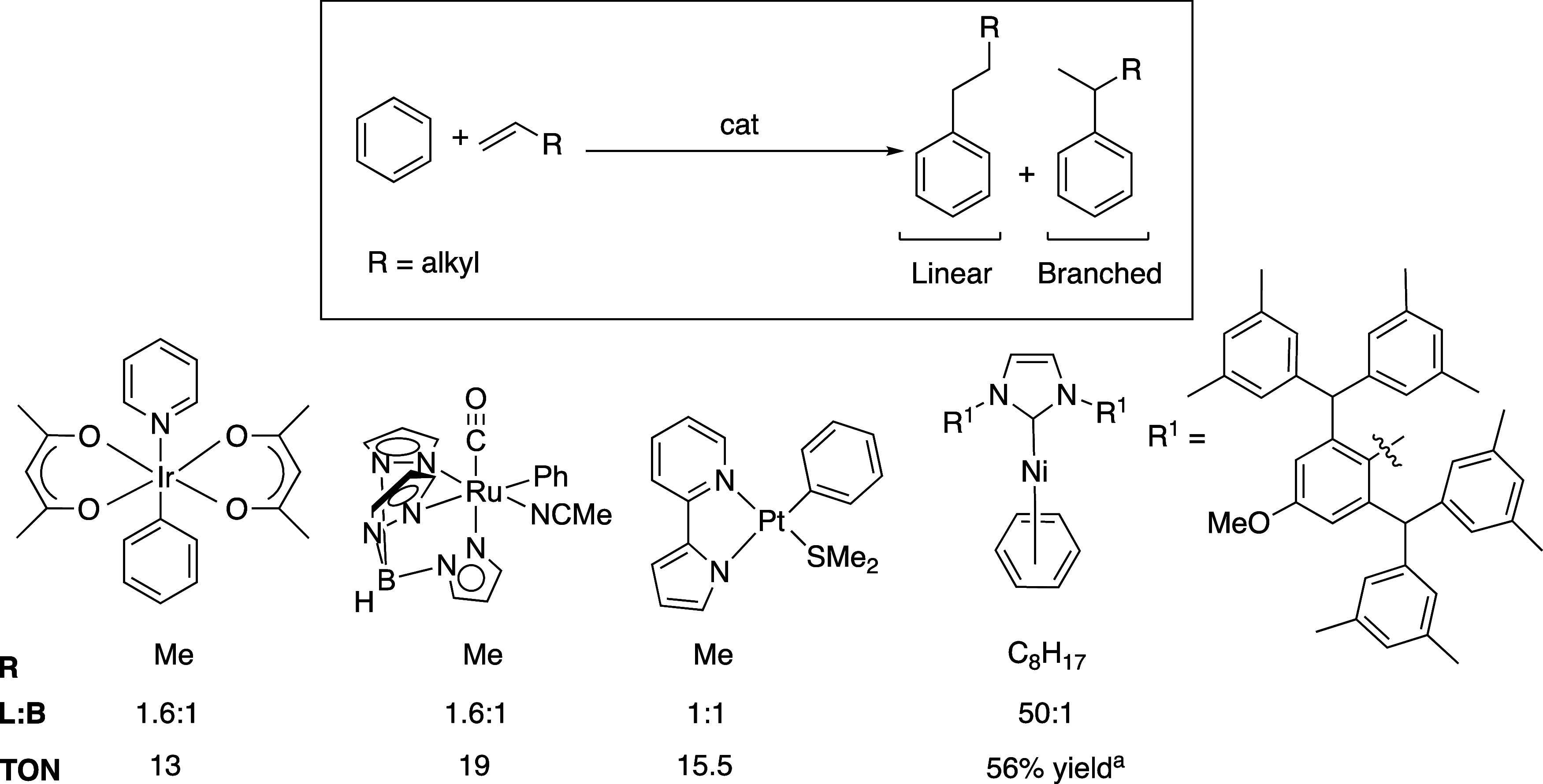
Catalysts for Arene Alkylation Using α-Olefins, and Some Examples of Turnover Numbers (TONs) and Linear/Branched Ratios (L/B). TON Refers to the Product/Catalyst Ratio after Catalyst Deactivation is Complete Yield based on olefins as the limiting reagent.

Our group and others have reported catalysts for arene alkylation using molecular Pt,^17−25^ Ru,^26−34^ Ir,^35,36^ and Ni complexes.^37,38^ With the exception of the Ni-mediated chemistry, the selectivity for linear alkyl arenes using α-olefins is modest, and, in the case of some Pt catalysts, the arene alkylation reactions are selective for branched products (Scheme 3).^17,25,27,37,39^ Catalysis based on Ru, Pt, and Ir were proposed to have similar mechanisms that involve olefin insertion into a M–aryl bond, arene coordination, and arene C–H activation to release an alkyl arene product.^40^ Some Pt catalysts are proposed to generate a Brønsted acid that initiates Friedel–Crafts catalysis.^41−43^ The Ni chemistry, reported by Hartwig, Eisenstein, and co-workers, is highly selective for linear alkyl arenes.^37,38^ The most selective Ni complex is (^*m*-Xyl^IPr^*OMe^)Ni(η^6^-C_6_H_6_).

**Scheme 4:**
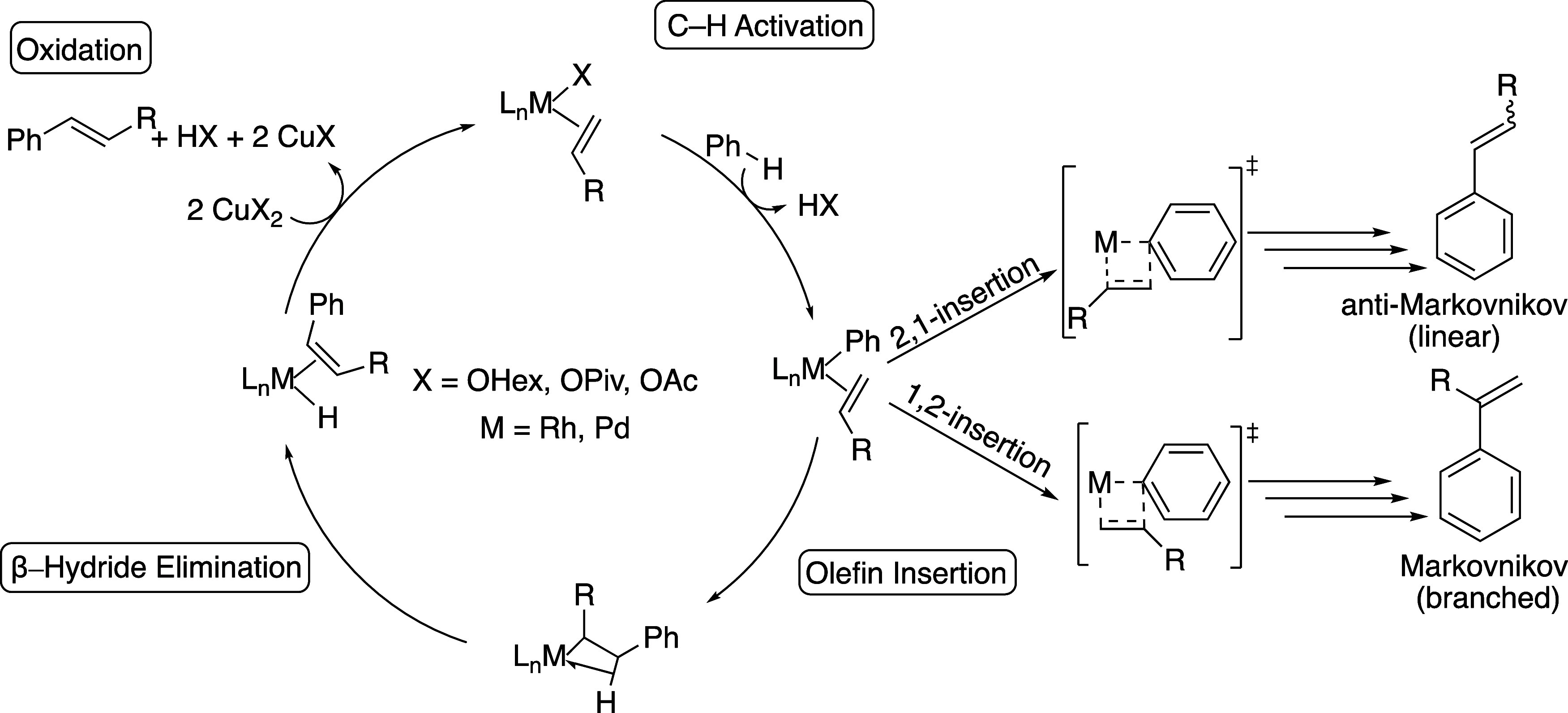
General Catalytic Cycle for Rh- and Pd-Catalyzed Arene Alkenylation Using Cu(II) Carboxylate Salts, with Benzene C–H Activation, Olefin Insertion, β-H Elimination, and an Oxidation of the M–H

In addition to catalytic arene alkylation using arenes and olefins, Ru,^29,44^ Rh,^15,16,45−50^ and Pd^51−54^ catalysts for single-step oxidative arene alkenylation have been reported. The general mechanisms reported for arene alkenylation using Rh and Pd include arene C–H activation, olefin insertion into a M–aryl bond, and product-forming β-H elimination, which is followed by the dissociation of alkenyl arenes. Generally, a M–H intermediate formed from the β-hydride elimination step reacts with an oxidant to re-form the starting catalyst through net H-atom abstraction. The selectivity of the products is determined in part by the olefin insertion, with a 2,1-insertion leading to the anti-Markovnikov (linear) product and the 1,2-insertion leading to the Markovnikov (branched) product (Scheme 4).^53,55^ These catalytic processes occur under Curtin–Hammett conditions for which the olefin insertions are reversible, and hence, product selectivity is based on the equilibria between olefin insertion products and the rate at which each proceeds to the final alkenyl arene product.^41^

**Scheme 5:**
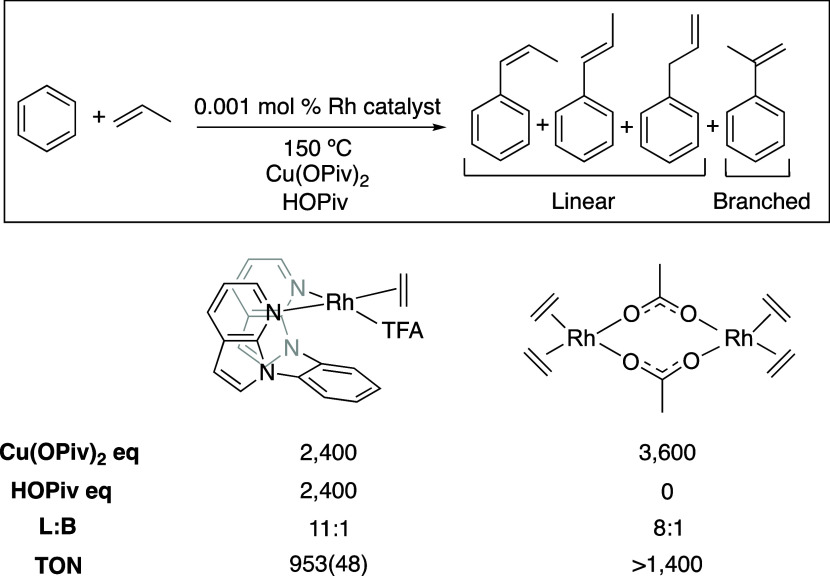
Examples of Selectivity for Linear Alkenyl Arenes Using Different Rh(I) Catalyst Precursors

For arene alkenylation using α-olefins, the Rh(I) dimer [Rh(μ-OAc)(C_2_H_4_)_2_]_2_ and (5-FP)Rh(TFA)(C_2_H_4_) (5-FP = 1,2-bis(*N*-7-azaindolyl)-benzene; TFA = trifluoroacetate) are selective for the linear products over the branched products (Scheme 5).^15,16^ The selectivity changes as a function of reaction conditions such as temperature, oxidant identity, and oxidant concentration with observed linear/branched (L/B) ratios between 6:1 and 18:1.^15,16^ Also, we studied arene alkenylation with multisubstituted olefins using [Rh(μ-OAc)(C_2_H_4_)_2_]_2_ as a catalyst precursor and Cu(II) carboxylate salts as the in situ oxidant.^49^ The selectivity for anti-Markovnikov products generally increases with steric bulk of the olefin, with vinyl cyclohexane giving a 27(1):1 anti-Markovnikov/Markovnikov product ratio and 1-butene giving a 7.7(6):1 anti-Markovnikov/Markovnikov product ratio. Additionally, monosubstituted olefins react faster than disubstituted olefins, and trisubstituted olefins are minimally reactive.

Herein, we report Ir-catalyzed propylene oxidative hydrophenylation using [Ir(μ-Cl)(coe)_2_]_2_ (coe = cyclooctene) as the catalyst precursor and CuX_2_ {X = OHex, OPiv, and OAc (OHex = 2-ethylhexanoate, OPiv = pivalate, OAc = acetate)} as the oxidant. Although low turnovers are observed, at optimized conditions, a L/B selectivity of 18:1 is observed at the start of the reaction, which increases to 42:1 after 42 h of reaction. The origin of the variation in the linear/branched selectivity as a function of reaction time was studied, and it was found to correlate with the ratio of Cu(II) to Cu(I) in the solution. Using this understanding, we demonstrate a high 65(3):1 linear/branched selectivity under one set of conditions. Based on previous studies of Rh and Pd catalysis,^51,56^ we propose that the change in linear/branched selectivity over time is the result of a change in catalyst speciation as Cu(II) carboxylate is consumed and Cu(I) carboxylate is formed.

## Results and Discussion

### Optimization of Reaction Conditions

**Scheme 6:**
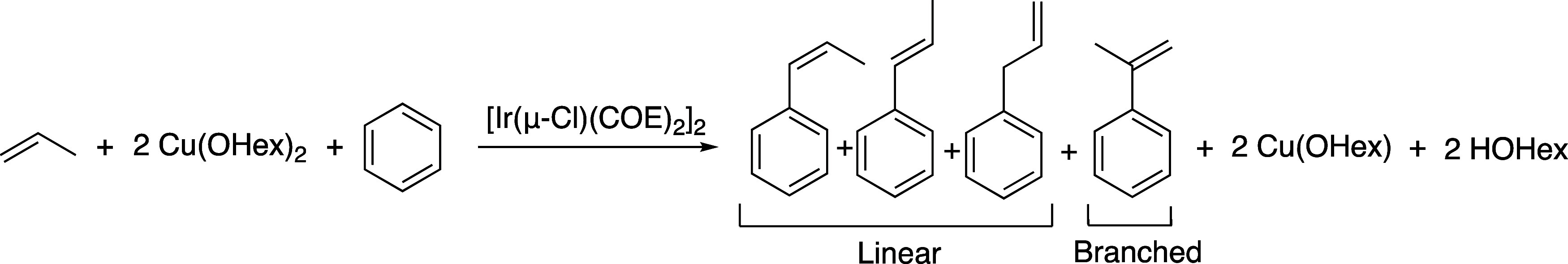
General Reaction Conditions, Showing the Production of Cu(I) and HOHex

We studied benzene alkenylation using [Ir(μ-Cl)(coe)_2_]_2_ as the catalyst precursor and propylene as the olefin. As shown in Scheme 6, the propenylbenzene products include *trans*-β-methylstyrene, *cis*-β-methylstyrene, allylbenzene, and α-methylstyrene. The hydrogenation of *trans*-β-methylstyrene, *cis*-β-methylstyrene, and allylbenzene would give 1-phenyl propane, and thus, they are considered linear products (i.e., anti-Markovnikov), and the hydrogenation of α-methylstyrene would produce cumene; thus, α-methylstyrene is the branched product (i.e., Markovnikov). Cu(II) carboxylates are the in situ oxidants and, under anaerobic conditions, are the limiting reagents. Thus, the reaction time was determined by a color change from blue to green and then to brown, which indicated the consumption of Cu(II). We have previously noted^15^ (see Figure S5 of the cited manuscript) the color change from blue for Cu(II) carboxylate salts to brown for Cu(I) carboxylate salts. The Cu(II) oxidant is necessary to sequester net 2 H atoms, and carboxylic acid is formed as a byproduct (Scheme 6). Therefore, the maximum yield under anaerobic conditions is 50% of Cu(OHex)_2_ added to the solution. We studied the turnovers (TOs) and linear/branched (L/B) ratios of the resulting propenylbenzenes from the benzene alkenylation reaction using propylene as the olefin, [Ir(μ-Cl)(coe)_2_]_2_ as the catalyst precursor, and varying amounts of Cu(OHex)_2_ and 2-ethylhexanoic acid (HOHex) at 150 °C. In order to optimize the reaction conditions, we quantified the impact of varying the amount of Cu(OHex)_2_ and HOHex.

Since the L/B ratio changes over the reaction time (see below), the L/B ratio reported in Table 1 is the highest L/B ratio recorded during the reaction time with different equivalents of HOHex. The TOs were within the deviation of the different amounts of acid (Table 1, entries 1–6). The L/B ratio remained statistically similar for all loadings of acid (Table 1, entries 2–6), except for a small increase in the L/B ratio with 0 equiv of acid used (Table 1, entry 1). The L/B ratio and TOs varied greatly depending on the loading of Cu(OHex)_2_. An increase in the amount of Cu(OHex)_2_ resulted in a higher L/B ratio and higher TOs (Table 1, entries 4 and 7–9). The TOs are low when the Cu(OHex)_2_ loading is decreased to 30 and 60 equiv, resulting in low yields with respect to Cu(OHex)_2_ (Table 1, entries 10 and 11), most likely due to the Ir-catalyzed Cu(OHex)_2_ decomposition being faster than the production of propenylbenzenes (we have identified a side reaction in which the Ir catalyst decomposes Cu(OHex)_2_; Figure S4). Thus, the low TOs of the propenylbenzene product relative to Cu(OHex)_2_ are likely the result of Ir-catalyzed decomposition of Cu(OHex)_2_.

**Table 1:**
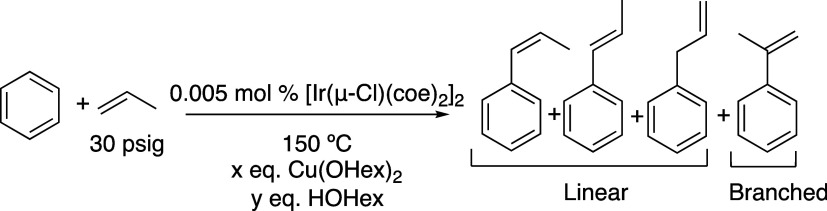
Optimization of Benzene Alkenylation Using Propylene^a^

entry	Cu(OHex)_2_ (equiv)	HOHex (equiv)	time (h)	TOs	highest L/B
1	240	0	42	11(1)	49(1):1
2	240	240	42	12(1)	42(3):1
3	240	480	42	14(1)	45(1):1
4	240	960	42	12(2)	42(2):1
5	240	1920	42	12(2)	44(1):1
6	240	3840	42	10(1)	41(2):1
7	120	960	12	2.8(4)	31(1):1
8	480	960	114	24(2)	52(5):1
9	960	960	246	49(3)	89(2):1
10	30	0	4	<1	n.q.
11	60	0	1	<1	n.q.

a Reaction conditions: 10 mL of benzene, 0.005 mol % of [Ir(μ-Cl)(coe)_2_]_2_, *x* equiv of Cu(OHex)_2_, *y* equiv of HOHex, 30 psig of propylene, and 150 °C. Catalyst loading is relative to that of benzene per single Ir atom. The turnovers (TO) of propenylbenzene products was quantified using GC-FID. Each data point represents the average of at least 3 independent reactions with the standard deviations shown. n.q.: insufficient TOs to calculate the L/B ratio.

**Figure 1:**
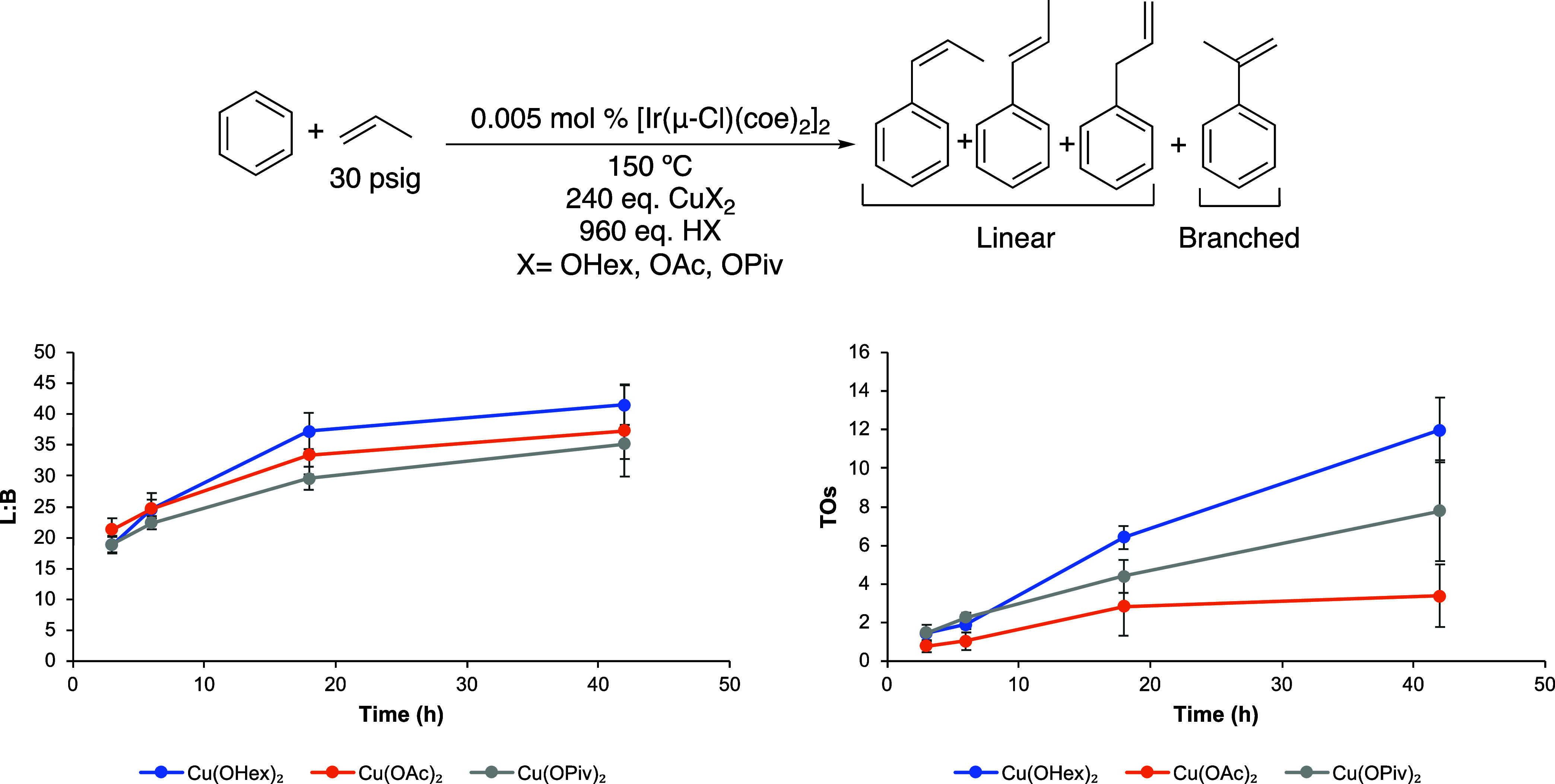
Linear/branched (L/B) vs time (left) and turnovers (TOs) vs time (right) plots of arene alkenylation using benzene and propylene as the olefin. TOs are based on the sum of all propenylbenzene products. Reaction conditions: 10 mL of benzene, 0.005 mol % of [Ir(μ-Cl)(coe)_2_]_2_, 240 equiv of CuX_2_ (X = OHex, OPiv, or OAc), 960 equiv of HX, 30 psig of propylene, and 150 °C. Catalyst loading is relative to benzene per single Ir atom. The turnover of propenylbenzenes is quantified using GC-FID. Each data point represents the average of at least 3 independent reactions with the standard deviations shown.

A variety of Cu(II) oxidants were tested including Cu(OHex)_2_, Cu(OPiv)_2_, Cu(OAc)_2_, and CuCl_2_ (Figure 1). The L/B ratios of the three Cu(II) carboxylate salts were statistically identical, while the TOs were not, most likely due to solubility issues of Cu(OAc)_2_. The reaction with CuCl_2_ did not produce propenylbenzenes.

**Figure 2:**
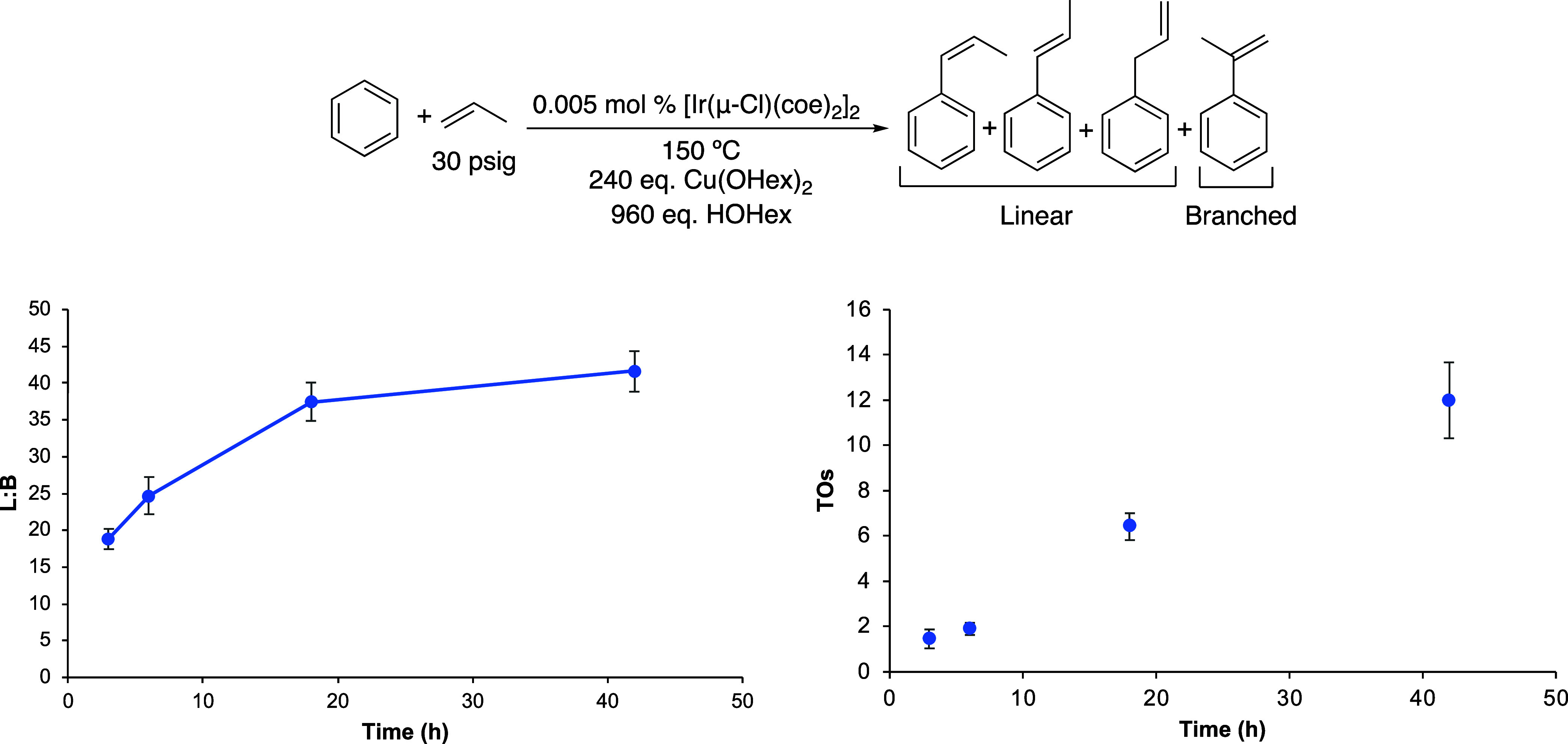
Linear/branched ratio (L/B) vs time (left) and turnovers (TOs) vs time (right) plots for benzene alkenylation using propylene as the olefin. The TOs are based on the sum of all propenylbenzene products. Reaction conditions: 10 mL of benzene, 0.005 mol % of [Ir(μ-Cl)(coe)_2_]_2_, 240 equiv of Cu(OHex)_2_, 960 equiv of HOHex, 30 psig of propylene, and 150 °C. Catalyst loading is relative to benzene per single Ir atom. The turnover of propenylbenzenes is quantified using GC-FID. Each data point represents the average of at least 3 independent reactions with the standard deviations shown.

For Ir-catalyzed benzene propenylation, we consistently observed a statistically significant increase in the L/B ratio over the reaction time (Figure 2). The conditions described in Table 1, entry 4, were selected as the standard conditions for benzene alkenylation using propylene as the olefin. Under anaerobic conditions with 10 mL of benzene, 0.005 mol % loading of [Ir(μ-Cl)(coe)_2_]_2_ (relative to benzene per single Ir atom), 240 equiv of Cu(OHex)_2_ (relative to Ir), and 960 equiv of HOHex at 150 °C, 12(2) TOs of propenylbenzenes were produced after 42 h. The L/B ratio was 18:1 after 3 h and increased to 42:1 after 42 h (Figure 2). Thus, we sought to understand the origin of the significant change in L/B selectivity. Three hypotheses are as follows: (1) isomerization of the branched product to linear products, (2) selective decomposition of the branched product under reaction conditions, or (3) a change in reaction conditions as the reaction proceeds that alters the anti-Markovnikov to Markovnikov selectivity.

To determine if α-methylstyrene is isomerized to linear products under catalytic conditions, 228(9) μmol of α-methylstyrene were added to a reaction with 10 mL of benzene, 0.005 mol % of [Ir(μ-Cl)(coe)_2_]_2_, 240 equiv of Cu(OHex)_2_, and 960 equiv of HOHex with 50 psig of ethylene and heated at 150 °C. Ethylene was used as the olefin in order to mimic catalysis under the standard conditions using propylene. The reaction was monitored using GC-FID, and after 42 h, the quantity of α-methylstyrene remained within the standard deviation, while *trans*-β-methylstyrene, *cis*-β-methylstyrene, or allylbenzene were not detected (Table 2). For this reaction, 8(1) TOs of styrene were observed, which confirmed that the catalytic conditions were achieved.

**Table 2:**
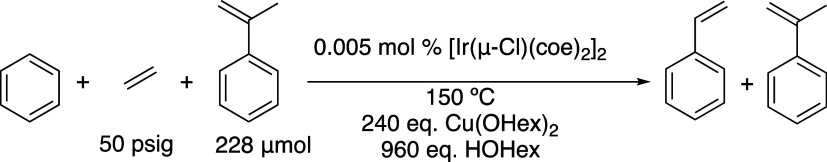
Testing for the Isomerization of α-Methylstyrene during Ir-Catalyzed Arene Alkenylation^a^

time (h)	α-methylstyrene (μmol)
0	228(9)
42	218(8)

a Reaction conditions: 10 mL of benzene, 228 μmol of α-methylstyrene, 0.005 mol % of [Ir(μ-Cl)(coe)_2_]_2_, 240 equiv of Cu(OHex)_2_, 960 equiv of HOHex, 50 psig of ethylene, and 150 °C. Catalyst loading is relative to benzene per single Ir atom. The turnover of α-methylstyrene is quantified using GC-FID. Each data point represents the average of at least 3 independent reactions with the standard deviations shown.

Since minimal allylbenzene was detected in the Ir-catalyzed benzene propenylation, we investigated whether allylbenzene could isomerize to *trans*-β-methylstyrene and *cis*-β-methylstyrene under catalytic conditions. When 223(14) μmol of allylbenzene were added to a reaction with 10 mL of benzene, 0.005 mol % loading of [Ir(μ-Cl)(coe)_2_]_2_, 240 equiv of Cu(OHex)_2_, and 960 equiv of HOHex with 50 psig of ethylene at 150 °C, there is only minor isomerization of the allylbenzene to *trans*-β-methylstyrene after 48 h of reaction (Table 3). Isomerization of allylbenzene to *trans*-β-methylstyrene was not observed in the absence of [Ir(μ-Cl)(coe)_2_]_2_ (Table S1). For this reaction, 6(1) TOs of styrene were produced, showing that catalytic conditions were achieved. This result suggests that allylbenzene is not formed and then subsequently isomerized to *trans*-β-methylstyrene when [Ir(μ-Cl)(coe)_2_]_2_ is used as the catalyst precursor.

**Table 3:**
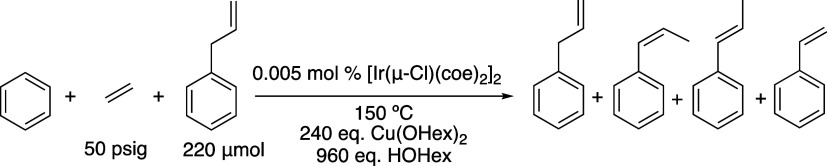
Testing for the Isomerization of Allylbenzene during Ir-Catalyzed Arene Alkenylation^a^

time (h)	allylbenzene (μmol)	α-methylstyrene (μmol)	*cis*-β-methylstyrene (μmol)	*trans*-β-methylstyrene (μmol)
0	223(14)	0	1(0)	8(2)
18	209(13)	0	2(0)	12(1)
48	200(13)	0	2(0)	16(1)

a Reaction conditions: 10 mL of benzene, 223 μmol of allylbenzene, 0.005 mol % of [Ir(μ-Cl)(coe)_2_]_2_, 240 equiv of Cu(OHex)_2_, 960 equiv of HOHex, 50 psig of ethylene, and 150 °C. Catalyst loading is relative to benzene per single Ir atom. The turnover of propenylbenzenes is quantified using GC-FID. Each data point represents the average of at least 3 independent reactions with the standard deviations shown.

**Figure 3:**
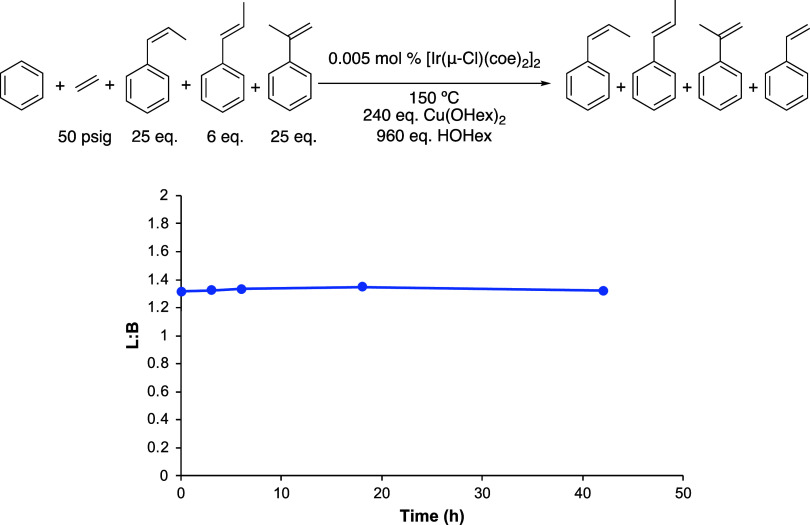
Linear/branched (L/B) vs time plot to test the selective decomposition of α-methylstyrene. Reaction conditions: 10 mL of benzene, 25 equiv of *trans*-β-methylstyrene, 6 equiv of *cis*-β-methylstyrene, 25 equiv of α-methylstyrene, 0.005 mol % of [Ir(μ-Cl)(coe)_2_]_2_, 240 equiv of Cu(OHex)_2_, 960 equiv of HOHex, 50 psig of ethylene, and 150 °C. Catalyst loading is relative to benzene per single Ir atom. The turnover of propenylbenzenes is quantified using GC-FID. Each data point represents the average of at least 3 independent reactions with the standard deviations, and deviations are too small to be seen on the graph.

**Figure 4:**
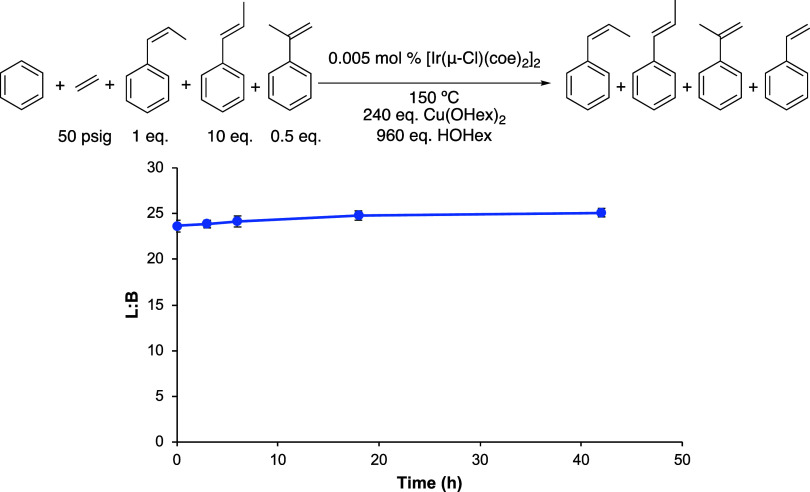
Linear/branched ratio (L/B) vs time plot to test the selective decomposition of α-methylstyrene. Reaction conditions: 10 mL of benzene, 10 equiv of *trans*-β-methylstyrene, 1 equiv of *cis*-β-methylstyrene, 0.5 equiv of α-methylstyrene, 0.005 mol % of [Ir(μ-Cl)(coe)_2_]_2_, 240 equiv of Cu(OHex)_2_, 960 equiv of HOHex, 50 psig of ethylene, and 150 °C. Catalyst loading is relative to benzene per single Ir atom. The turnover of propenylbenzenes is quantified using GC-FID. Each data point represents the average of at least 3 independent reactions with the standard deviations shown.

Product stability under catalytic conditions was studied to determine if α-methylstyrene selectively decomposed, which would increase the L/B ratio. Previously, we found that when using RhCl_3_ as the catalyst precursor and dioxygen as the oxidant, the L/B increased during the reaction.^47^ The increase in L/B over time during aerobic Rh catalysis was found to be the result of the selective decomposition of α-methylstyrene under the specific reaction conditions. To study if there is a selective decomposition of products using [Ir(μ-Cl)(coe)_2_]_2_ as a catalyst precursor, quantities of *trans*-β-methylstyrene, *cis*-β-methylstyrene, and α-methylstyrene were added to 10 mL of benzene with a 0.005 mol % loading of catalyst with 240 equiv of Cu(OHex)_2_ and 960 equiv of HOHex with 50 psig of ethylene. Upon heating to 150 °C, the L/B ratio of this starting solution and the concentrations of each substrate remained unchanged over time (Figure 3). This reaction was repeated with a 24:1 L/B ratio in order to better model the product distribution under standard catalytic reaction conditions, which remained invariant over 42 h of heating at 150 °C, and the concentration of the substrates remained the same (Figure 4). These results suggest that the increase in L/B as a function of time is not likely the result of the selective decomposition of α-methylstyrene.

As the Ir-catalyzed benzene propenylation reaction progresses, there is an accumulation of propenylbenzene products and 2 equiv of both Cu(I) and HOHex per TO of the propenylbenzene product. Additionally, side products including biphenyl and phenyl-2-ethylhexanoate (PhOHex) are accumulated over time (Figure S5). We speculated that the changes in reaction conditions as a result of side product and byproduct formation could result in a change in the active catalyst structure or reaction pathway that could alter the L/B selectivity.

**Figure 5:**
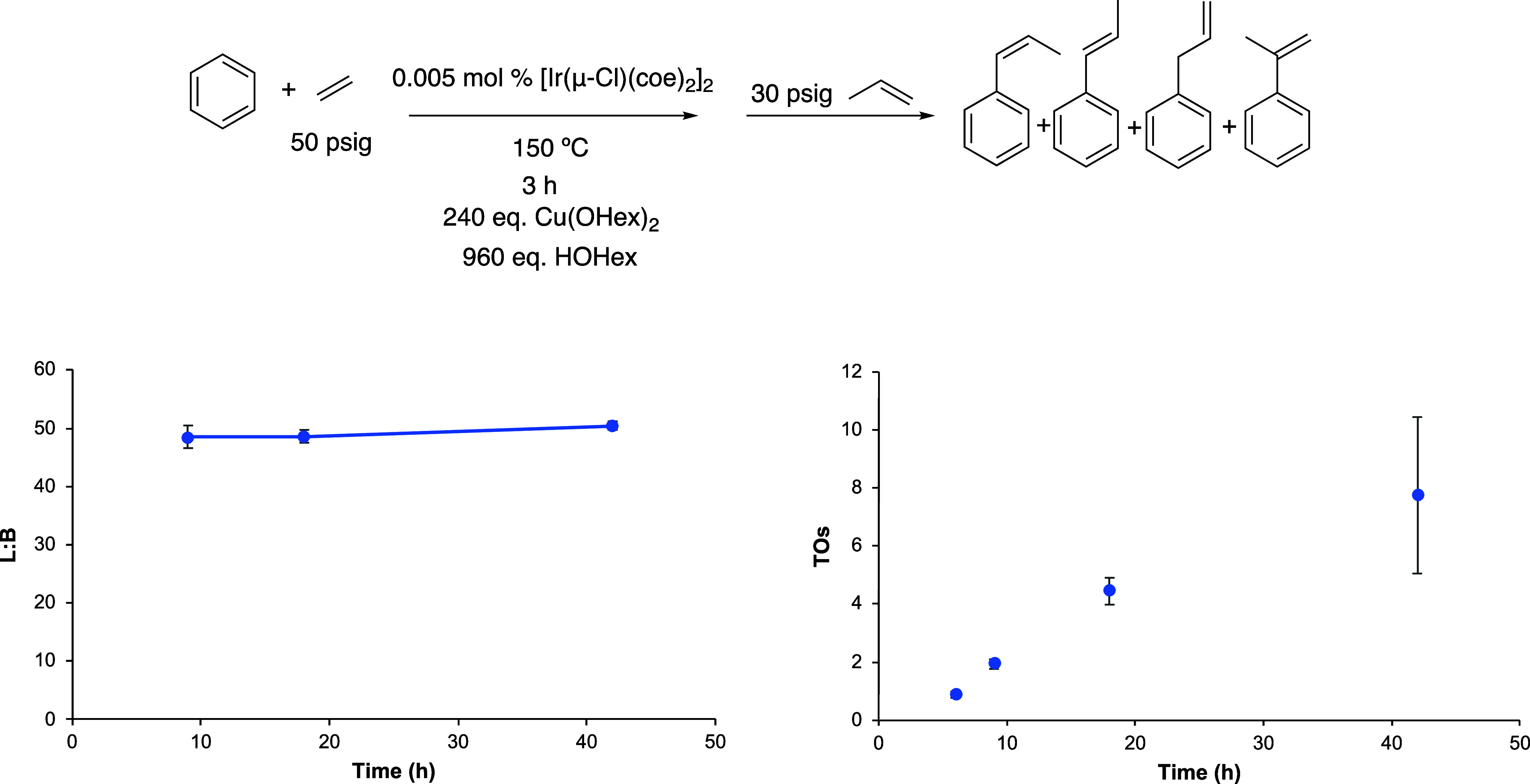
Linear/branched ratio (L/B) vs time (left) and turnovers (TOs) of propenylbenzene vs time plot (right). TOs are based on the sum of all propenylbenzene products. Reaction conditions: 10 mL of benzene, 0.005 mol % of [Ir(μ-Cl)(coe)_2_]_2_, 240 equiv of Cu(OHex)_2_, 960 equiv of HOHex, 50 psig of ethylene, heat at 150 °C for 3 h, then purge out ethylene, add 30 psig propylene, and heat. Catalyst loading is relative to benzene per single Ir atom. The turnover of propenylbenzenes is quantified using GC-FID. Each data point represents the average of at least 3 independent reactions with the standard deviations shown.

To determine whether the change in the L/B selectivity for propylene oxidative hydrophenylation is caused by a change in the reaction conditions, all components of the reaction, except propylene, were heated. These sets of reactions were first heated with ethylene, and after 3 h, ethylene was removed with a dinitrogen purge. Then, propylene was added to the reactor, and the reactor was heated. A set of reactions was performed with 10 mL of benzene, 0.005 mol % of [Ir(μ-Cl)(coe)_2_]_2_, 240 equiv of Cu(OHex)_2_, 960 equiv of HOHex, 50 psig of ethylene and heated at 150 °C for 3 h. The TOs of styrene after 3 h {1.2(3) TOs} were similar to those of propenylbenzenes {1.4(4)} under the standard conditions after 3 h. Then, ethylene was purged out of the reactor, 30 psig of propylene were added, and the reactor was heated at 150 °C. After 9 h of reaction time (3 h with ethylene and 6 h with propylene), the L/B ratio (48(2):1) was much higher than under standard conditions where only propylene was used as the olefin (∼28:1) (Figure 5). Additionally, the 48(2):1 ratio remained constant over the 20 h reaction period. This finding suggests that a change in the reaction conditions that occurs as a result of arene alkenylation is likely responsible for the change in the L/B ratio. This experiment also provides further evidence against the change in L/B selectivity being the result of α-methylstyrene decomposition.

**Figure 6:**
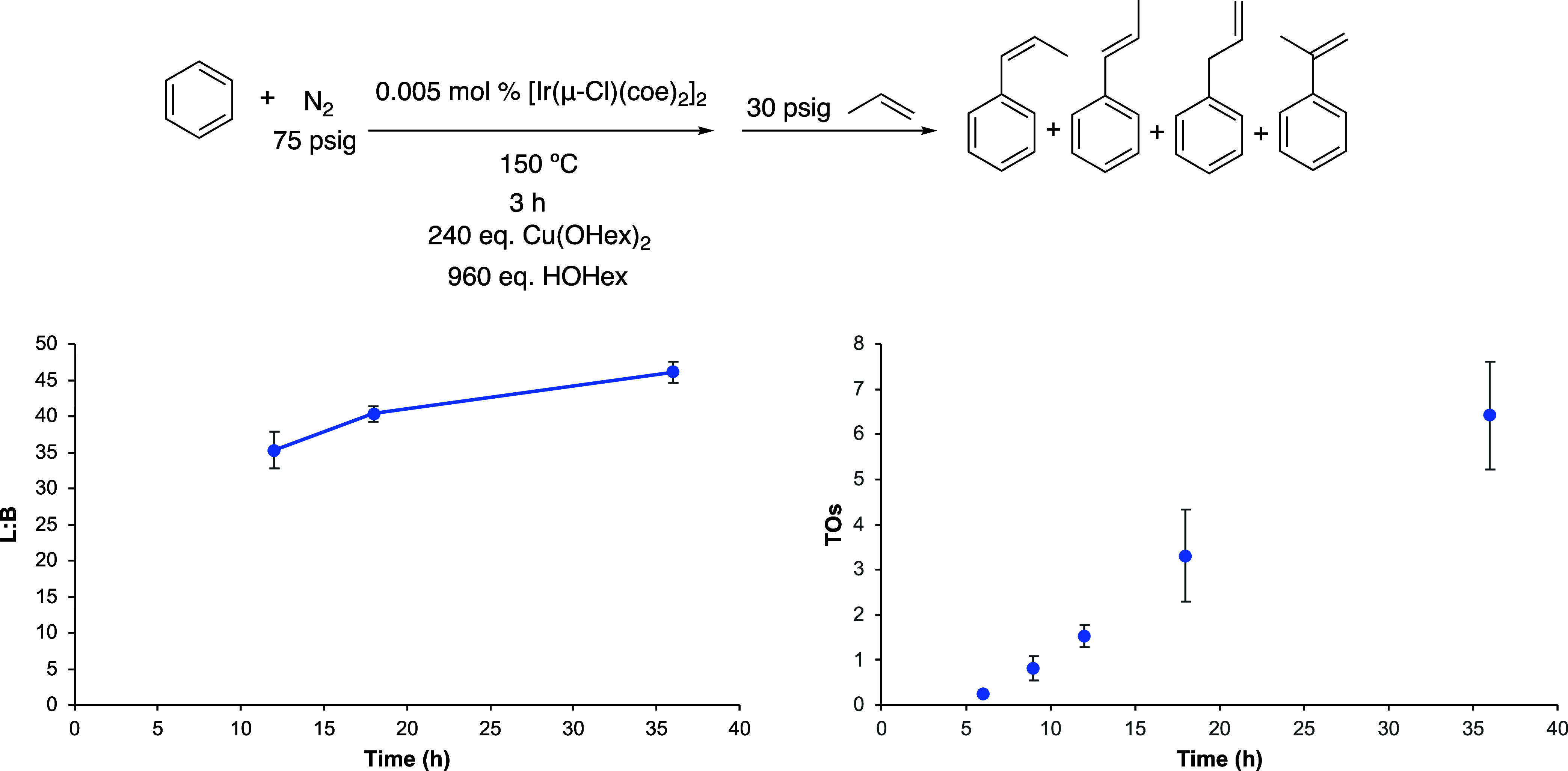
Linear/branched ratio (L/B) vs time (left) and turnovers (TOs) of propenylbenzene vs time (right) plot. TOs are based on the sum of all propenylbenzene products. Reaction conditions: ethylene then propylene 10 mL of benzene, 0.005 mol % of [Ir(μ-Cl)(coe)_2_]_2_, 240 equiv of Cu(OHex)_2_, 960 equiv of HOHex, 75 psig of dinitrogen, heat at 150 °C for 3 h, then release the pressure, add 30 psig propylene, and heat at 150 °C. Catalyst loading is relative to benzene per single Ir atom. The turnover of propenylbenzenes was quantified using GC-FID. Each data point represents the average of at least 3 independent reactions with the standard deviations shown.

After the change in the initial L/B ratio was observed upon heating with ethylene first (Figure 5), the reaction was repeated with an otherwise identical preheating step without any olefin. A set of reactions was performed with 10 mL of benzene, 0.005 mol % of [Ir(μ-Cl)(coe)_2_]_2_, 240 equiv of Cu(OHex)_2_, and 960 equiv of HOHex. These mixtures were heated under 70 psig of N_2_ at 150 °C for 3 h prior to the addition of propylene. After the addition of propylene, the reactions were sampled, and the L/B ratio and TOs were quantified. As shown in Figure 6, the L/B selectivity as a function of time is nearly statistically identical under standard conditions. The color of these reactions is still blue, suggesting a near quantitative amount of Cu(II). The TOs for the reactions using N_2_ first and then propylene are slightly lower than those under standard conditions, which could be due to catalyst deactivation or Cu(II) carboxylate decomposition after heating for 3 h.

As shown in Scheme 4, for each equiv of propenyl arene produced, 2 equiv of the Cu(II) oxidant are required. The consumption of Cu(OHex)_2_ produces Cu(OHex) and HOHex. Under the standard conditions reported above, the reactions are performed with an excess of acid. As the reaction proceeds, both Cu(OHex) and HOHex are accumulated. In addition to Cu(OHex) and HOHex, side products, including PhOHex and biphenyl, are observed. We studied whether the addition of each of these components at the beginning of the reaction results in a change in the initial L/B selectivity as well as any change over the course of the reaction (Figure S6).

**Figure 7:**
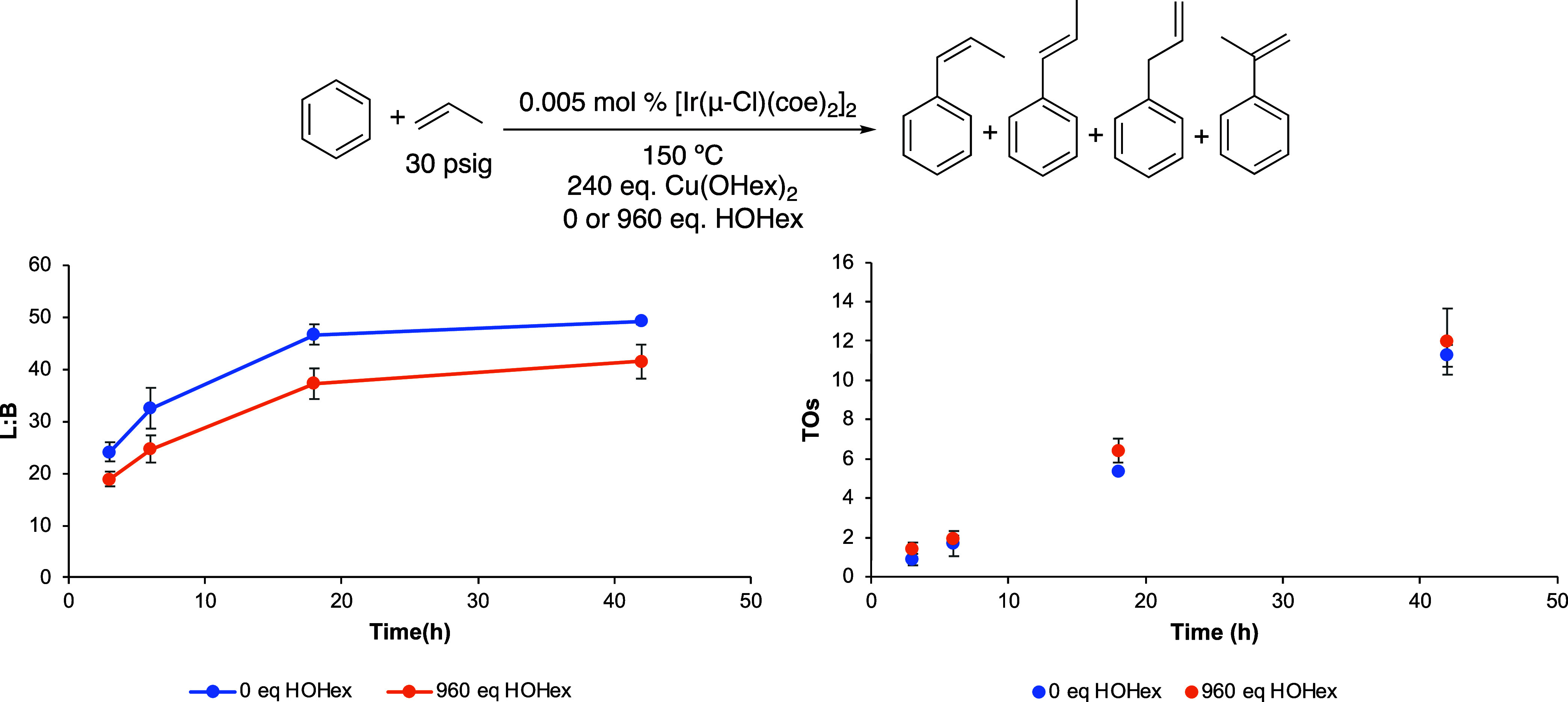
Linear/branched ratio (L/B) vs time (left) and turnovers (TOs) vs time (right) plot for 0 or 960 equiv of HOHex. TOs are based on the sum of all propenylbenzene products. Reaction conditions: 10 mL of benzene, 0.005 mol % of [Ir(μ-Cl)(coe)_2_]_2_, 240 equiv of Cu(OHex)_2_, 0 or 960 equiv of HOHex, 30 psig of propylene, and 150 °C. Catalyst loading is relative to benzene per single Ir atom. The turnover of propenylbenzenes is quantified using GC-FID. Each data point represents the average of at least 3 independent reactions with the standard deviations shown.

The effect of HOHex was studied with a set of reactions containing 10 mL of benzene, 0.005 mol % of [Ir(μ-Cl)(coe)_2_]_2_, 240 equiv of Cu(OHex)_2_, and 30 psig of propylene at 150 °C. These data were compared to those obtained under the standard conditions (Figure 2) with 960 equiv of HOHex. The reaction with 0 equiv of HOHex exhibits a slightly higher L/B ratio after 42 h (49(1):1) vs conditions with 960 equiv of acid (42(3):1) (Figure 7). The TOs vs time in the presence vs absence of HOHex are statistically the same. A significant difference between reactions in the presence and absence of HOHex is that the consumption of Cu(OHex)_2_ occurs faster in the absence of HOHex. This is partly due to the production of the phenyl ester, PhOHex, the rate of which has an inverse dependence rate on the HOHex concentration.^16,57^ Under the conditions with no added HOHex, 7(1) equiv of PhOHex were produced relative to Ir after 42 h, while the conditions with 960 equiv of acid gave 1.0(2) equiv of PhOHex relative to Ir after 42 h.

**Figure 8:**
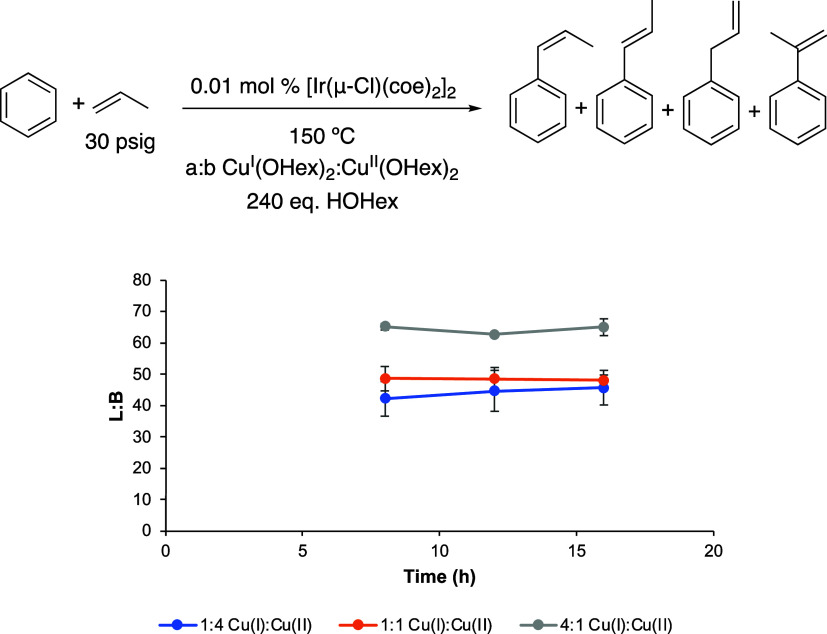
Linear/branched ratio (L/B) vs time plot for the comparison of different approximate Cu(I)/Cu(II) ratios. Reaction conditions: 10 mL of benzene, 0.01 mol % of [Ir(μ-Cl)(coe)_2_]_2_, a/b of Cu(I)/Cu(II), 240 equiv of HOHex, 30 psig of propylene, and 150 °C. Catalyst loading is relative to benzene per single Ir atom. The turnover of propenylbenzenes is quantified using GC-FID. Each data point represents the average of at least 3 independent reactions with the standard deviations shown.

Next, the effect of the amount of Cu(I) was studied. For all reactions described herein, Cu(I) carboxylate was generated in situ with 10 mL of benzene, 0.01 mol % loading of [Ir(μ-Cl)(coe)_2_]_2_, varied equivalents of Cu(OHex)_2_, and 240 equiv of HOHex. The reactions were pressurized with 75 psig of N_2_ and heated at 150 °C until the color changed. After the solutions turned brown, presumably indicating near quantitative Cu(I) formation, varied amounts of Cu(II) were added to the reactors to give different Cu(II) to Cu(I) ratios. When a 1:1 ratio of Cu(I)/Cu(II) was used with 60 equiv of Cu(I), 60 equiv of Cu(II), and 30 psig of propylene at 150 °C, the L/B ratio of 48(2):1 remained constant within the standard deviation from 6 to 18 h. When a 1:4 Cu(I)/Cu(II) ratio was used, the L/B ratio was 45(6):1 at 18 h, while with a 4:1 Cu(I)/Cu(II) ratio, the L/B ratio was 65(3):1 at 18 h (Figure 8). These results indicate that the Cu(II)/Cu(I) ratio is important for controlling the L/B ratio with a higher amount of Cu(I) in the solution leading to a higher L/B ratio.

**Scheme 7:**
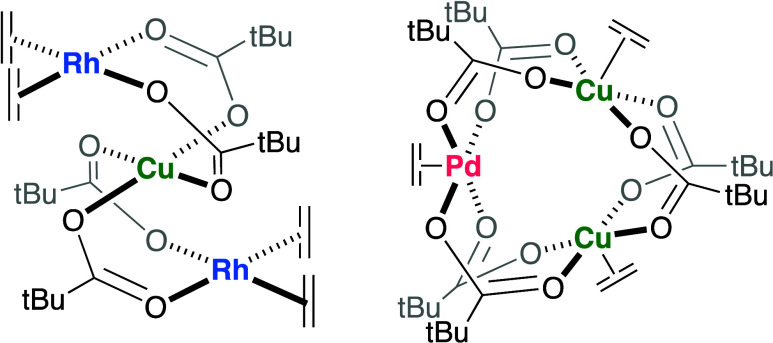
Mixed Rh/Cu and Pd/Cu Species that are Likely the Active Catalysts for Styrene Production^51,56^ Reproduced from ref (52) Copyright 2023 American Chemical Society.

Previously, we studied the catalyst speciation for arene alkenylation using Rh(I) and Pd(II) precursors and a combination of experimental and density functional theory (DFT) calculations. We propose that mixed metallic Rh/Cu and Pd/Cu species are likely active catalysts for styrene production (Scheme 7).^51,56^ Importantly, although our studies demonstrate that the structures shown in Scheme 7 are likely the most active catalysts for the conversion of benzene, ethylene, and Cu(II) carboxylate to styrene, we also propose that other mixed metallic species are viable catalysts.^52^ Thus, there is likely more than one active catalyst and the actual catalyst(s) can evolve with reaction conditions. Thus, for the Ir-catalyzed conversion of benzene, propylene, and Cu(II) carboxylate, for which the L/B ratio is highly dependent on the ratio of Cu(II) to Cu(I), we believe that it is reasonable to propose that the active Ir catalyst(s) are mixed metallic Ir/Cu species. The various accessible Ir/Cu species are likely readily interconverted, and the dominant form likely varies with the Cu(II)/Cu(I) ratio. Unfortunately, attempts to grow crystals of mixed Ir/Cu species were unsuccessful.

## Summary and Conclusions

**Scheme 8:**
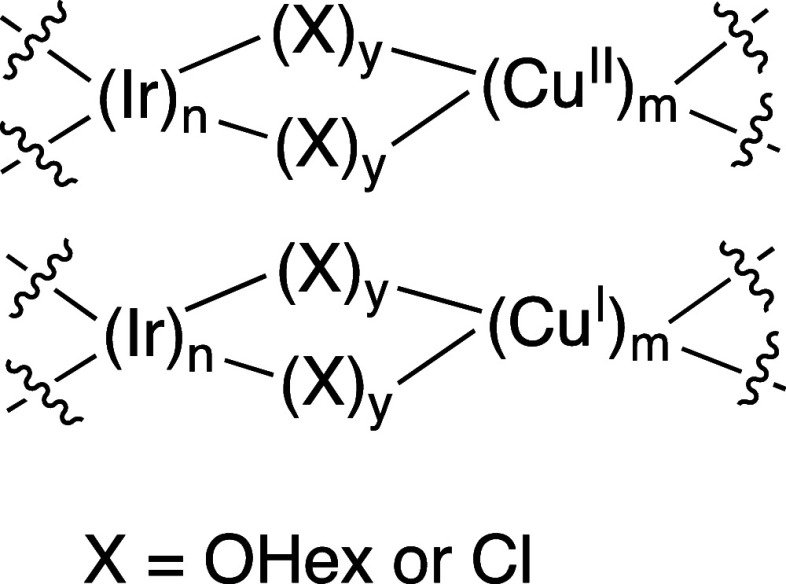
Generic Representation of Possible Mixed Metallic Ir/Cu Complexes that could Serve as Catalysts for Arene Alkenylation

We have shown that [Ir(μ-Cl)(coe)_2_]_2_ is a catalyst precursor for benzene alkenylation using propylene as an olefin at 150 °C. Our major observations are as follows: (1) As the reaction progresses, there is a significant change in the L/B selectivity with a L/B ratio of 18:1 at 3 h and a L/B ratio of 42:1 at 42 h under one set of conditions. (2) The change in the L/B ratio is not due to the isomerization or consumption of α-methylstyrene. (3) The change in the ratio is most likely due to an increase in the amount of Cu(I) in the solution as the reaction progresses. When a higher amount of Cu(I) is present in solution, the L/B ratio is higher and remains constant. (4) Using an approximate Cu(I)/Cu(II) ratio of 4:1, we observe a highly anti-Markovnikov selective catalytic process with a L/B ratio of 65(3):1. (5) Based on previous studies of similar catalysis using Rh and Pd, we propose that mixed metallic Ir/Cu species (Scheme 8) that readily interconvert under catalytic conditions are possible active catalysts for propenylbenzene production, and that catalysts with Cu(I) in the structure are more selective for anti-Markovnikov products than mixed metallic species with Cu(II). With the current experimental data, we cannot definitely eliminate from consideration the possibility of an in situ-formed heterogeneous catalyst. However, the lack of observed induction periods as well as the consistent change in linear/branched ratios seem to be most consistent with a homogeneous catalyst. Further, the proposed homogeneous mixed metallic Ir/Cu catalyst is consistent with our previous evidence for mixed metallic Rh/Cu and Pd/Cu catalysts for oxidative arene alkenylation.^51,52,56^

In the absence of more well-defined structures of Ir/Cu catalytic species, it is difficult to explain the significant changes in anti-Markovnikov/Markovnikov selectivity. Previously, for the proposed mixed metallic Rh/Cu catalyst for the oxidative conversion of benzene and ethylene,^56^ we have suggested that structural changes upon incorporation of Cu(II) into the active catalyst might facilitate benzene coordination and C–H activation. If similar Ir/Cu species are the active catalysts for the conversion of benzene and propylene reported herein, it would not be surprising that the replacement of Cu(II) with Cu(I) would alter the catalyst structure in a manner that could significantly impact the relative rate of 1,2- vs 2,1-propylene insertion and, hence, impact the linear/branched selectivity.

## Experimental Section

### General Considerations

Unless otherwise noted, all reactions were performed under an inert atmosphere in a dinitrogen-filled glovebox. Glovebox purity was maintained by periodic dinitrogen purges to ensure that the O_2_ concentration was below 25 ppm. Benzene was dried by using a solvent purification system with an activated alumina column. Ethylene and propylene (99.9%) were purchased in gas cylinders from Linde Gas and Equipment and used as received. All other reagents were purchased from commercial sources and used as received. GC-FID was performed using a Shimadzu GC-2014 instrument with a 30 m × 0.32 mm DB-5MS UI column with a 0.25 μm film thickness. Turnovers were quantified by linear regression analysis of the gas chromatograms using standard samples of the authentic products purchased from a commercial source. A plot of peak area ratios vs molar ratios gave a regression line using hexamethylbenzene as an external standard. The slopes and correlation coefficients of the regression lines for the following compounds were as follows: allylbenzene (1.39, 0.999), α-methylstyrene (1.24, 0.999), *cis*-β-methylstyrene (1.48, 0.999), *trans*-β-methylstyrene (1.38, 0.999), biphenyl (0.96, 0.999), phenyl-2-ethylhexanonate (1.03, 0.999), and styrene (1.78, 0.999).

### Synthesis of [Ir(μ-Cl)(coe)_2_]_2_

A previous procedure was followed with modifications.^58^ IrCl_3_·*x*H_2_O (0.5 g, 1.4 mmol) was added to a Fisher Porter reactor with isopropanol (7.5 mL), H_2_O (23 mL), and coe (1.5 mL). The reactor was pressurized with 75 psig of N_2_ and heated at 85 °C for 1.5 h. The orange solid was collected and washed with cold ethanol to yield 0.4198 g of [Ir(μ-Cl)(coe)_2_]_2_ (0.47 mmol, yield = 70%).

### General Procedure for Propenylbenzene Production

A stock solution of [Ir(μ-Cl)(coe)_2_]_2_ (2.8 mmol, 0.005 mol % of Ir relative to benzene) was added to oven-dried Astraglass Innovations Fisher Porter reactors inside of a dinitrogen-filled glovebox. Each Fisher Porter sample was charged with Cu(OHex)_2_ (0.4703 g, 1.34 mmol), HOHex (0.42 mL, 2.63 mmol), and benzene (10 mL, 112 mmol). The vessels were sealed, pressurized with propylene (30 psig), stirred, and heated at 150 °C. The reactions were allowed to cool to room temperature and sampled under N_2_ after withdrawing 200 μL aliquots. The reactors were repressurized with gases and then reheated. The aliquots were washed with saturated sodium carbonate, and a hexamethylbenzene stock solution was added (0.182 mg, 1.12 μmol). The aqueous and organic layers were separated, and the organic layers were analyzed using GC-FID.

### Production of Cu(I)

Inside a dinitrogen-filled glovebox, a starting amount of Cu(OHex)_2_ (0.1177 g, 0.33 mmol; 0.2354 g, 0.67 mmol; or 0.4706 g, 1.34 mmol) was added to each Fisher Porter reactor. HOHex (0.21 mL, 1. 33 mmol), [Ir(μ-Cl)(coe)_2_]_2_ (2.8 mmol, 0.005 mol % of Ir relative to benzene), and benzene (10 mL, 112 mmol) were added to the reactor. The reactors were sealed and pressurized with 75 psig of N_2_. The reactors were placed in a 150 °C oil bath and were stirred and heated until a color change to brown was observed, assuming quantitative production of Cu(I). After cooling, the reactors were vented to ambient pressure and then brought back into the glovebox. Cu(OHex)_2_ was added back to the reactor (0.1177 g, 0.33 mmol; 0.2354 g, 0.67 mmol; or 0.4706 g, 1.34 mmol) in order to give an approximate ratio of Cu(I) to Cu(II). The reactors were sealed, removed from the glovebox, and pressurized with 30 psig of propylene. The reactors were heated and stirred at 150 °C. To analyze these reactions, the procedure described in the general procedure for propenylbenzene production was followed.

## Supporting Information Available

Representative GC-FID chromatograms; calibration curves; plots for product vs time for catalytic reactions, and NMR spectra and additional experimental details (PDF)
