# Supplementary material for: Highly Anti-Markovnikov Selective Oxidative Arene Alkenylation Using Ir(I) Catalyst Precursors and Cu(II) Carboxylates
Source: Organometallics. 2024 Mar 20;43(7):774–86. doi: 10.1021/acs.organomet.4c00030 (PMC11005047; doi:10.1021/acs.organomet.4c00030)
Supplement: Supplementary file 1 — om4c00030_si_001.pdf [file om4c00030_si_001.pdf]

Supporting Information for

Arene Alkenylation using Ir(I) Precursors: Investigation of Regioselectivity using α-Olefins

Hannah E. Ketcham, [†] Weihao Zhu, [†] and T. Brent Gunnoe[†*]

[†] Department of Chemistry, University of Virginia, Charlottesville, VA 22904;

Table of Contents:

Representative GC-FID chromatogram for propylene oxidative arene alkenylation……..………S3

Calibration curves for allyl benzene, **α**-methylstyrene, cis-β-methylstyrene, trans-β-methylstyrene, biphenyl, phenyl-2-ethylhexanonate, styrene …………………………....S4

TOs for each propenyl benzene product under standard conditions……………….…..S5

Evidence for $[Ir(\mu\text{-}Cl)(COE)_2]_2$ catalyzed Cu(II) decomposition………………...…………S5

Production of side products biphenyl and PhOHex under standard conditions……..….S5

L:B and TO vs Time with 0 and 16 equiv of PhOHex……………………………………...S6

L:B and TO vs Time using $[Ir(\mu\text{-}Cl)(coe)_2]_2$ and $[Ir(\mu\text{-}Cl)(C_2H_4)_2]_2$…………………….…….S6

Synthesis of $[Ir(\mu\text{-}OPiv)(coe)_2]_2$……………………………………………………………S7

^{1}H NMR studies of $[Ir(\mu\text{-}OPiv)(coe)_2]_2$, $Cu(OPiv)_2$, and ethylene at 120 ºC………..……S8

Concentration of allylbenzene without the addition of $Ir(\mu\text{-}Cl)(coe)_2]_2$……………..…….S8

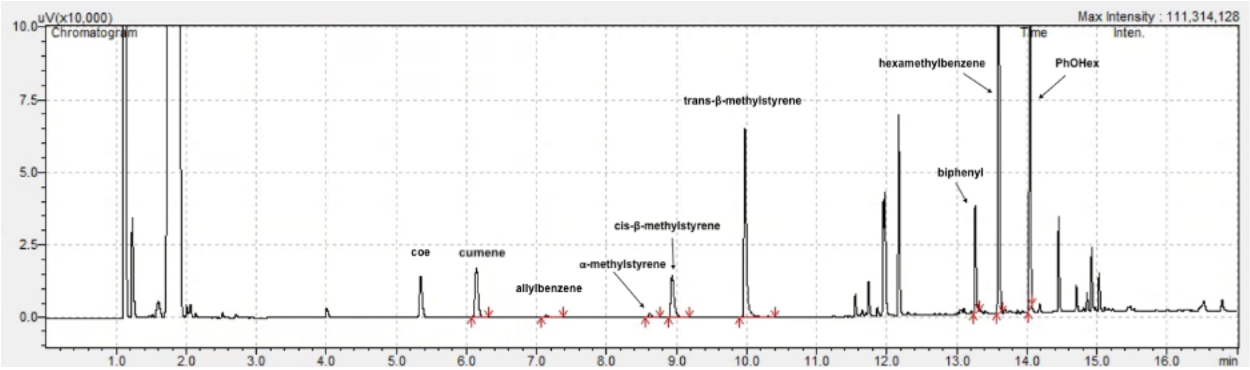

Figure S1. Representative GC-FID chromatogram for propylene oxidative arene alkenylation

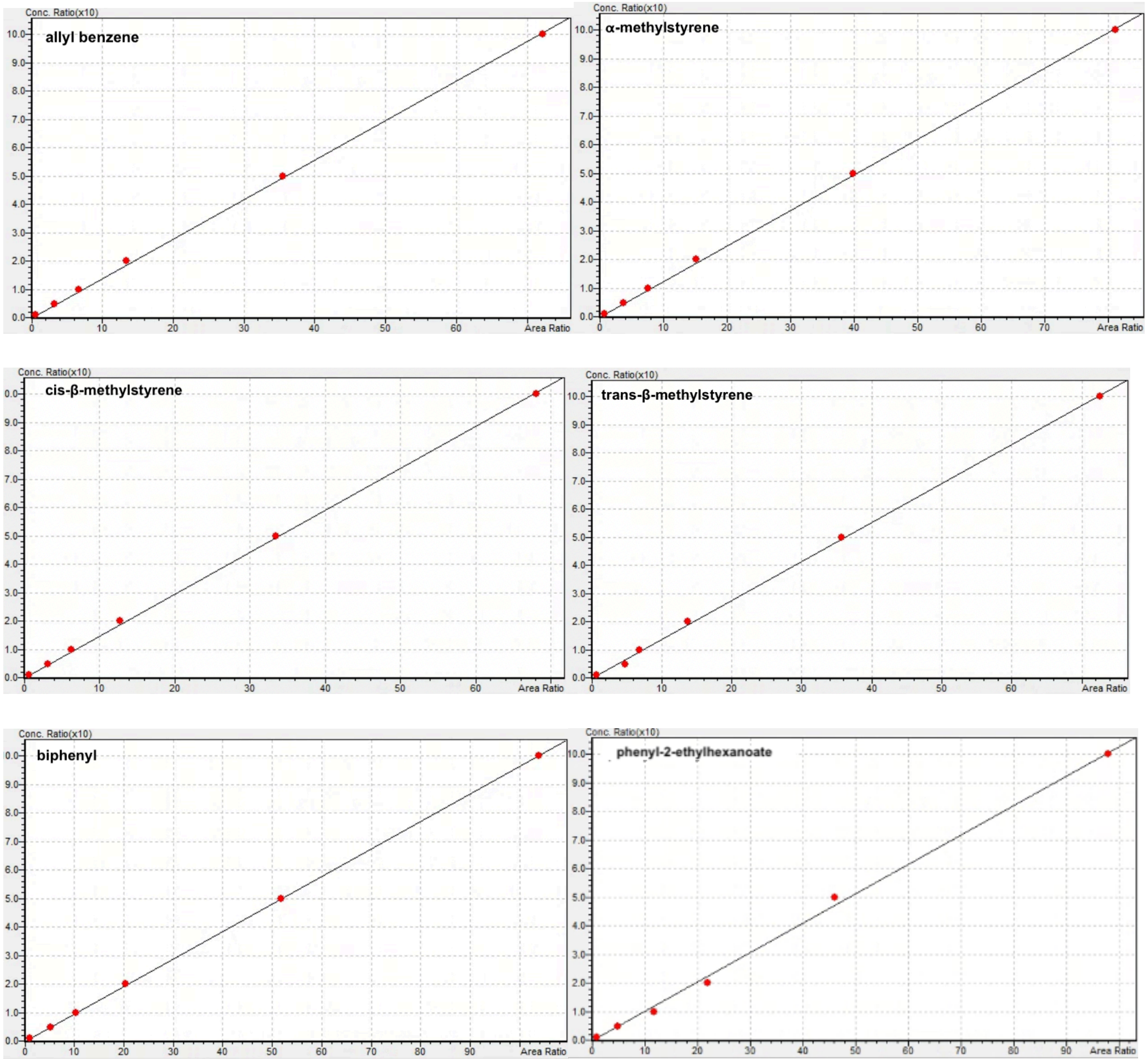

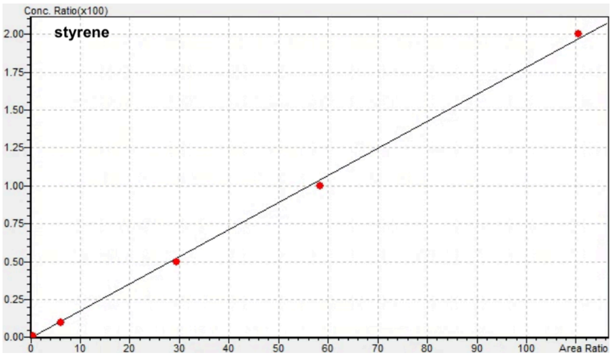

Figure S2. Calibration curves for allyl benzene, **α**-methylstyrene, cis-β-methylstyrene, trans-β-methylstyrene, biphenyl, phenyl-2-ethylhexanoate, styrene

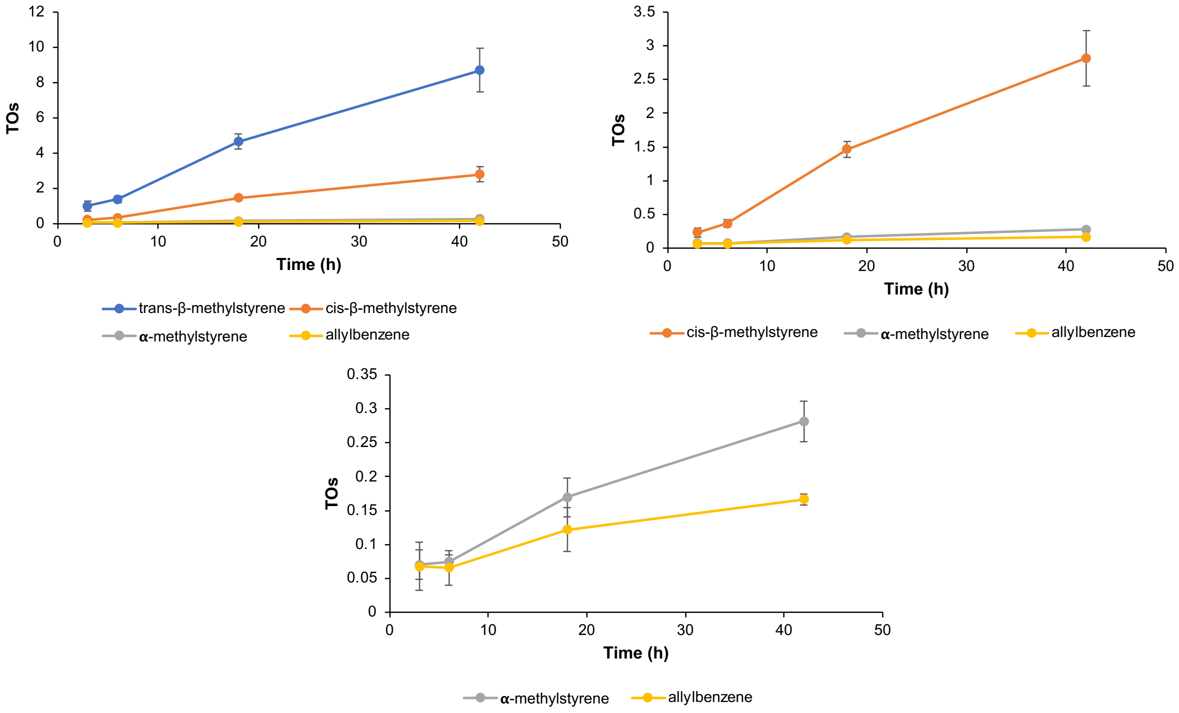

Figure S3. TOs for each propenyl benzene product under standard conditions

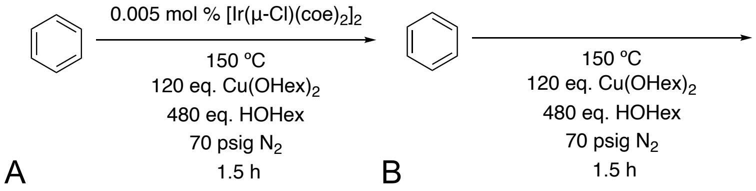

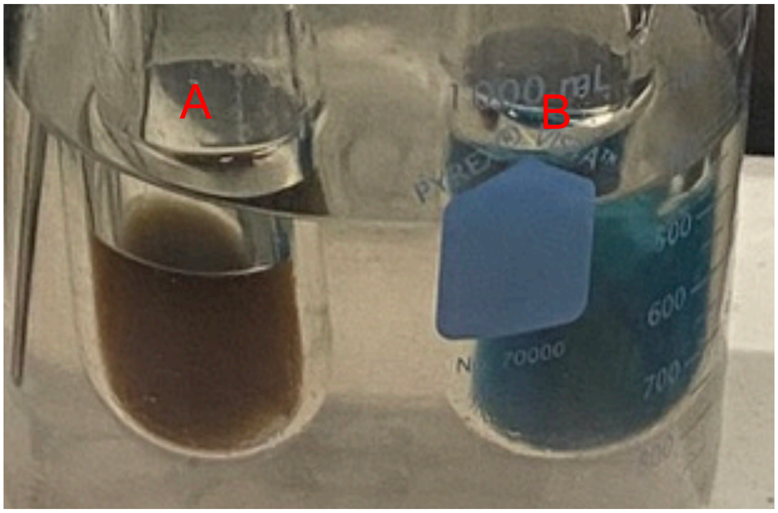

Figure S4. Evidence for $[Ir(\mu\text{-}Cl)(coe)_2]_2$ catalyzed Cu(II) decomposition. Reaction conditions: Reaction A: 10 mL benzene, 0.005 mol % $[Ir(\mu\text{-}Cl)(coe)_2]_2$, 120 equiv $Cu(OHex)_2$, 480 equiv HOHex, 75 psig N_2, 150 ºC, 1.5 h. Reaction B: 10 mL benzene, 120 equiv $Cu(OHex)_2$, 480 equiv HOHex, 75 psig N_2, 150 ºC, 1.5 h. Color change to brown of reaction A demonstrates the decomposition of the Cu(II) in the presence of $[Ir(\mu\text{-}Cl)(coe)_2]_2$.

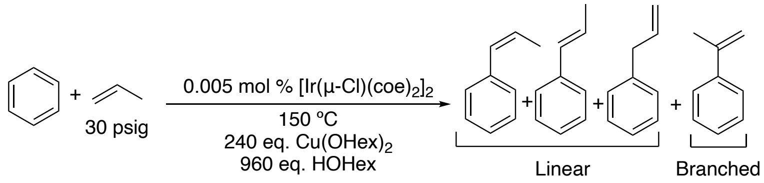

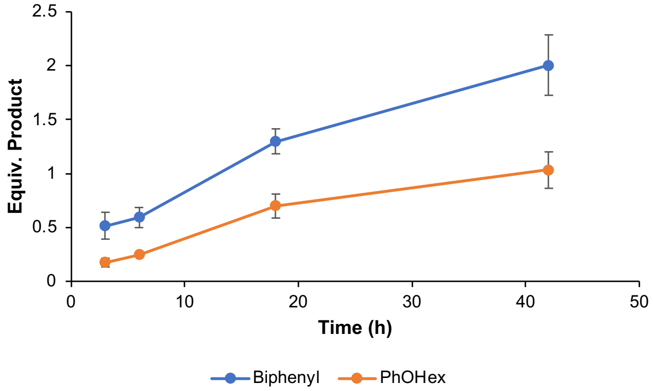

Figure S5. Equiv product vs time plot of side products (biphenyl and PhOHex) of benzene alkenylation under standard conditions. Reaction conditions: 10 mL benzene, 0.005 mol % $[Ir(\mu\text{-}Cl)(coe)_2]_2$, 240 equiv $Cu(OHex)_2$, 960 equiv HOHex, 30 psig propylene, 150 ºC. Catalyst loading is relative to benzene per single Ir atom. Turnovers were quantified using GC-FID. Each data point is the average of at least 3 independent reactions with standard deviations shown.

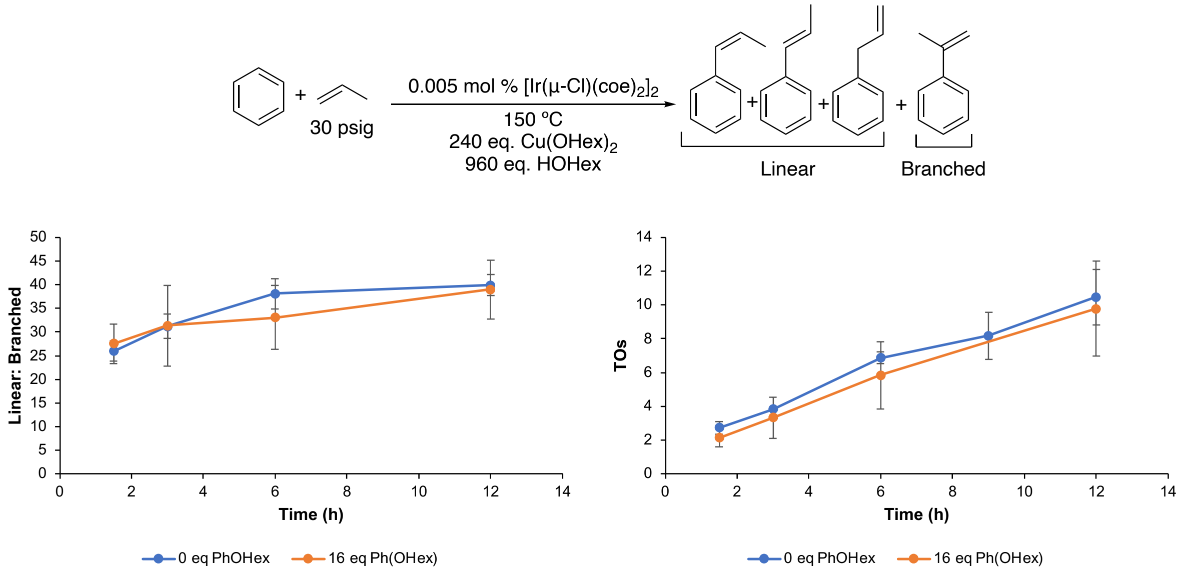

Figure S6. Linear:branched ratio (L:B) versus time (left) and turnovers (TOs) versus time (right) plots for addition of phenyl 2-ethylhexanoate. Reaction conditions: 0.005 mol% Ir ($[Ir(\mu\text{-}Cl)(coe)_2]_2$, 480 equiv $Cu(OHex)_2$, 1920 equiv HOHex, 30 psig propylene, 150 ºC. Catalyst loading is relative to benzene per single Ir atom. Turnovers of propenyl benzenes were quantified using GC-FID. Each data point is the average of at least 3 independent reactions with standard deviations shown.

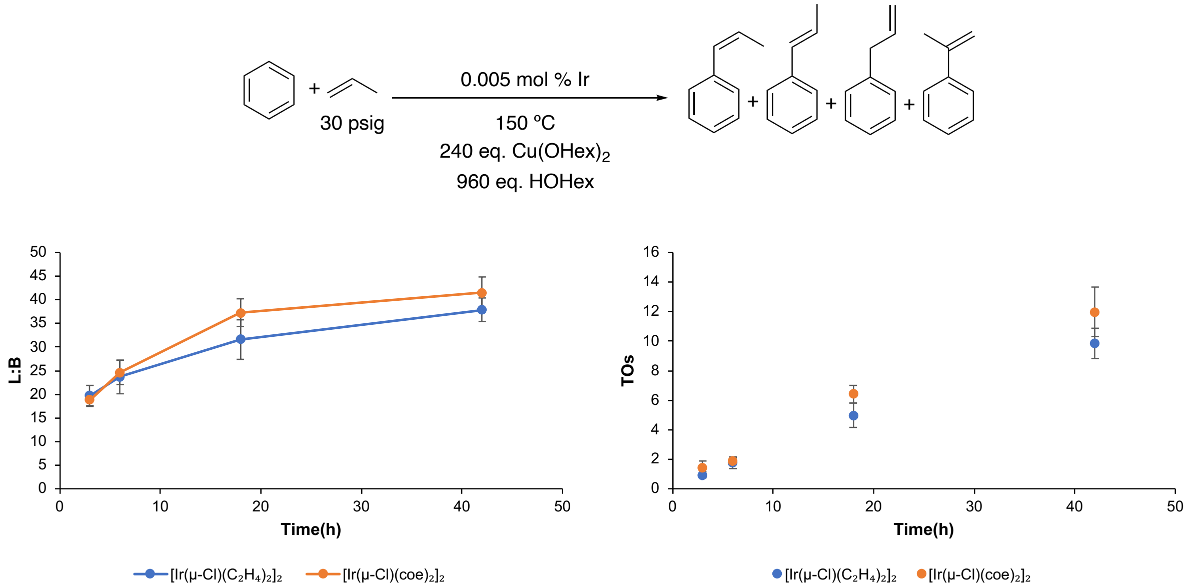

Figure S7. Linear:branched ratio (L:B) versus time (left) and turnovers (TOs) versus time (right) plots for comparison of Ir(I) catalyst precursors. Reaction conditions: 0.005 mol% Ir ($[Ir(\mu\text{-}Cl)(coe)_2]_2$ or $[Ir(\mu\text{-}Cl)(C_2H_4)_2]_2$), 240 equiv $Cu(OHex)_2$, 960 equiv HOHex, 30 psig propylene, 150 ºC. Catalyst loading is relative to benzene per single Ir atom. Turnovers of propenyl benzenes were quantified using GC-FID. Each data point is the average of at least 3 independent reactions with standard deviations shown.

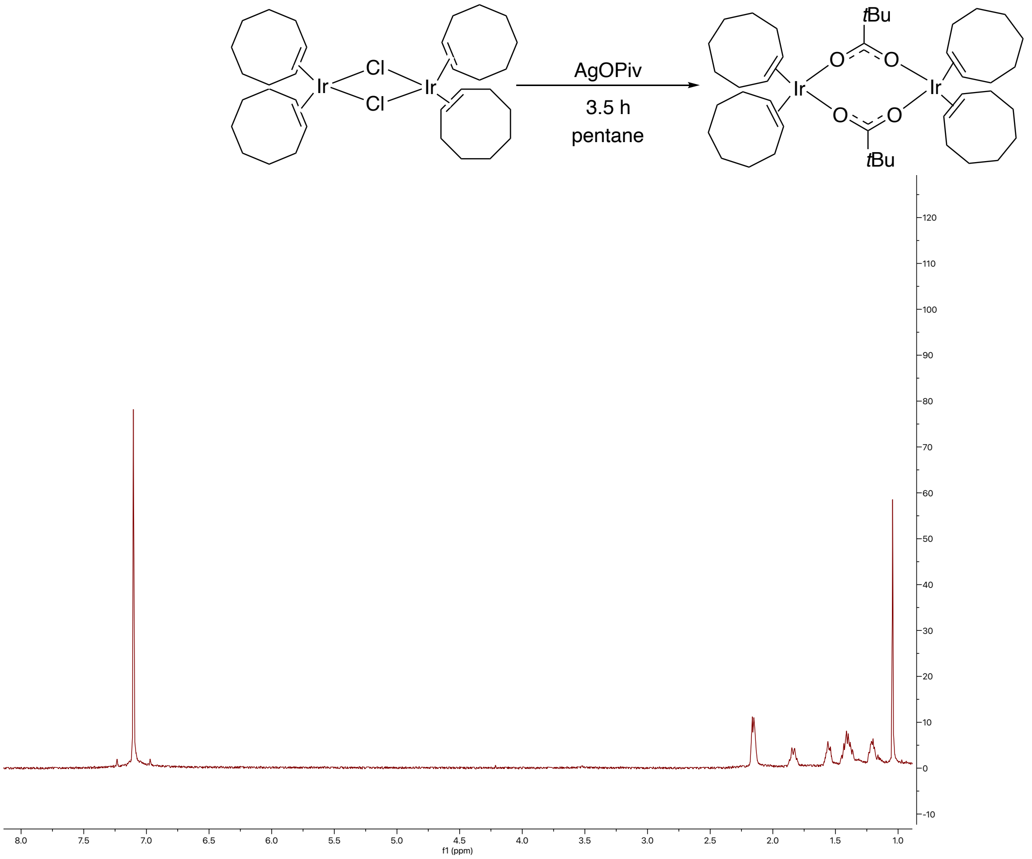

Figure S8. Synthesis of [Ir(μ-OPiv)(coe)$_2$]$_2$.

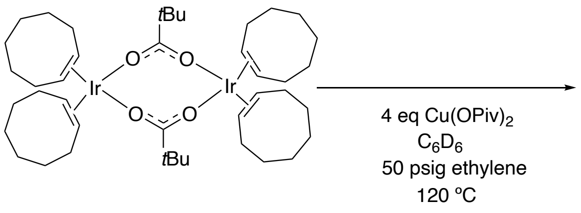

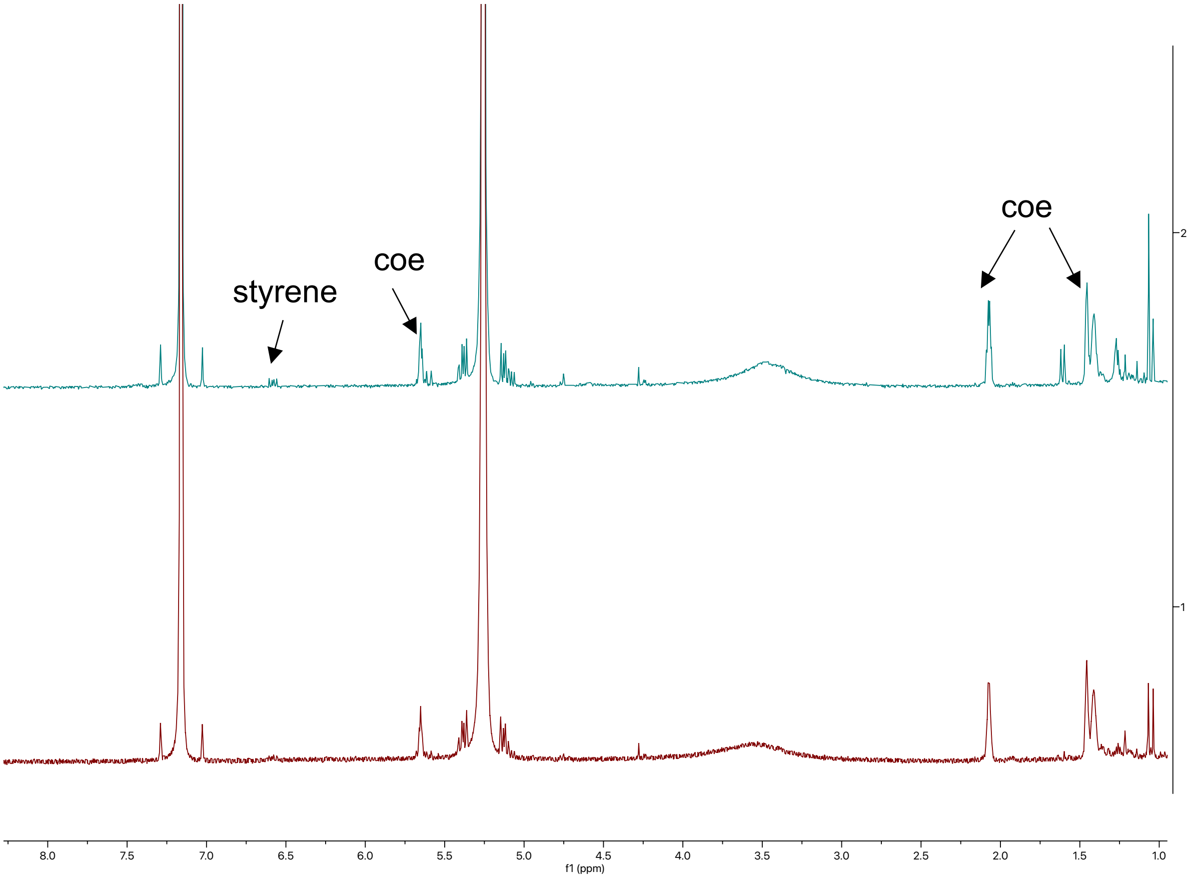

Figure S8. $[Ir(\mu\text{-}OPiv)(coe)_2]_2$, 4 equiv $Cu(OPiv)_2$, 50 psig ethylene at 120 ºC. Bottom spectra is after 10 minutes of heating, top is after 2 h of heating at 150 ºC.

Table S1. Control reaction testing for the isomerization of allylbenzene without $[Ir(\mu\text{-}Cl)(coe)_2]_2$

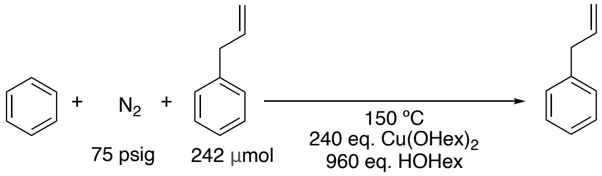

Time (h)	allylbenzene (μmol)	α-methylstyrene (μmol)	*cis*-β-methylstyrene (μmol)	*trans*-β-methylstyrene (μmol)
0	242(34)	0	0	0
48	252(32)	0	0	0
